# Synovial tissue macrophages: friend or foe?

**DOI:** 10.1136/rmdopen-2017-000527

**Published:** 2017-12-06

**Authors:** Mariola Kurowska-Stolarska, Stefano Alivernini

**Affiliations:** 1 Institute of Infection, Immunity and Inflammation, University of Glasgow, Glasgow, UK; 2 Rheumatoid Arthritis Pathogenesis Centre of Excellence (RACE), Universities of Glasgow, Birmingham and Newcastle, Glasgow, Birmingham and Newcastle, UK; 3 Institute of Rheumatology, Fondazione Policlinico Universitario A Gemelli, Catholic University of the Sacred Heart, Rome, Italy

**Keywords:** synovitis, inflammation, arthritis

## Abstract

Healthy synovial tissue includes a lining layer of synovial fibroblasts and macrophages. The influx of leucocytes during active rheumatoid arthritis (RA) includes monocytes that differentiate locally into proinflammatory macrophages, and these produce pathogenic tumour necrosis factor. During sustained remission, the synovial tissue macrophage numbers recede to normal. The constitutive presence of tissue macrophages in the lining layer of the synovial membrane in healthy donors and in patients with RA during remission suggests that this macrophage population may have a role in maintaining and reinstating synovial tissue homeostasis respectively. Recent appreciation of the different origins and functions of tissue-resident compared with monocyte-derived macrophages has improved the understanding of their relative involvement in organ homeostasis in mouse models of disease. In this review, informed by mouse models and human data, we describe the presence of different functional subpopulations of human synovial tissue macrophages and discuss their distinct contribution to joint homeostasis and chronic inflammation in RA.

**Key messages**

- There are different macrophage subpopulations in the human synovium with the potential to contribute either to joint homeostasis or to chronic inflammation in rheumatoid arthritis (RA).
- Experimental models of arthritis support the concept that synovial tissue macrophages have a pivotal role in reinstating joint homeostasis.
- Deeper understanding of the role of human synovial tissue macrophages in the regulation of joint homeostasis offers the prospect of new therapeutic strategies for RA.

## Synovial tissue homeostasis

Synovial joints link the musculoskeletal system, and the joint synovium facilitates movement. Bones are linked by the joint capsule and collagenous ligaments, and the bone surface is covered with articular cartilage that absorbs impact. The joint capsule consists of an outer fibrous membrane containing ligaments and proprioceptive sensory nerves that regulate the posture and motion of the joint and an inner synovial membrane that produces synovial fluid that lubricates the joint during movement and nourishes avascular cartilage. In healthy people, this complex joint structure is self-regulated by homeostatic mechanisms to maintain well-functioning articulation, and the synovial membrane is vital for maintaining joint homeostasis.^1^ In rheumatoid arthritis (RA), this normal joint structure is progressively compromised due to inflammation of the synovial membrane that fails to resolve, and this ultimately leads to loss of joint function.

The synovial membrane is a highly specialised, multifunctional structure consisting of two layers: the first is a thin (eg, <2 mm in radiocarpal joints by ultrasound imaging)^2^ but highly cellular lining layer composed of two cell types: synovial fibroblasts and synovial macrophages,^1^ and the second is a supporting sublining layer containing loose connective tissue with sublining fibroblasts, a rich network of sympathetic and sensory nerves and blood and lymphatic vasculature that provides oxygen/nutrients and immune-drainage.^1^ Synovial fibroblasts provide the extracellular matrix (ECM) that supports the structure of the synovium and secrete hyaluronic acid and lubricin into the synovial fluid.^1^ Synovial tissue macrophages (STM) are constitutively resident in healthy synovium. Their tissue-specific function remains unresolved, but this likely includes sentinel joint homeostasis. Other immune cells (lymphocytes, mast cells and dendritic cells) are scarce in the normal synovium and localise mainly in perivascular areas of the sublining layer.^3^

In patients with RA, the synovial membrane becomes hypertrophic (eg, 2–5 mm in radiocarpal joints)^2^ due to synovial fibroblast proliferation, increased blood/lymphatic vasculature and an inflammatory influx of immune cells from the circulation. The products of these activated cells lead to destruction of cartilage and bone, pain and loss of joint function.^4^ These changes appear consequential to aberrant homeostatic mechanisms that fail to resolve inflammatory synovitis. *Understanding synovial homeostasis may offer the prospect of new therapeutic strategies for RA.*

## Synovial tissue inflammation and resolution in RA

RA is the most common inflammatory arthropathy, affecting approximately 0.4 million people in the UK,^5^ causing morbidity and reduced quality of life and represents a substantial socioeconomic and healthcare burden. An interplay between genetics (>100 loci), environmental factors (eg, cigarette smoking and occupational exposure to silica) and alterations in the mucosal microbiome can contribute to the development of RA.^6 7^ Clinical RA is preceded by an asymptomatic preclinical breach of tolerance to citrullinated self-peptides, activation of autoreactive T cells and production of anticitrullinated peptide-specific antibodies (ACPA). Joint injury or other poorly defined events can then lead to activation of the synovial tissue vasculature permitting localisation of ACPA immune complexes in the synovium. These complexes activate synovial tissue fibroblasts and bone cells (osteoclasts) and perpetuate the influx of leucocytes, for example, neutrophils, monocytes, Th1/Th17 and B cells. These cells are activated by local stimuli (eg, endogenous Toll-like receptors (TLR) ligands, proinflammatory cytokines and hypoxia), further modulated by genetic and epigenetic modifications of gene expression to propagate the synovitis. Depending on the dominant cellular component and distinct molecular pathogenic pathways, RA synovitis can be characterised as myeloid (macrophage rich), lymphoid (containing T/B cell follicles) and fibroid (leucocyte low) 4 8 (*comprehensive reviews on mechanisms and type of synovitis*). Emerging data suggest that this underlying cellular heterogeneity in RA may influence the clinical outcome to therapies targeting different biological pathways.^9^

Recent advances in RA therapies mainly target the mediators of inflammation (eg, tumour necrosis factor (TNF), interleukin (IL)-6R and janus kinase (JAKs)), or block the adaptive immune response (eg, T cell stimulation or B cell function). These therapies are life-long, expensive and offer inadequate responses in 30%–50% of patients with RA.^7 10^ Furthermore, of those who respond, approximately half will relapse within months of treatment cessation and few achieve long-term remission with restoration of joint function.^10 11^ Remission of RA is defined by sustained resolution of swollen and painful joints and by normalised biomarkers of inflammation. These include erythrocyte sedimentation rate, which contributes to a low disease activity score of 44 joints of less than 1.6,^12^ and resolution of synovial inflammation confirmed by normal blood flow (by Power Doppler ultrasound).^2 11^ Early remission can stop the substantial cartilage loss and rebalance bone turnover, which are key in recovering joint function.^11 13 14^ The fact that some patients sustain drug-free remission is proof of concept that immunological homeostasis and healthy joint remodelling can be reinstated in RA. Therefore, understanding homeostatic mechanisms in these patients could help to develop therapeutic strategies to reinstate synovial homeostasis in RA. Experimental models of arthritis support the concept that STMs have a pivotal role in this process.^15^

## STM subpopulations

The functions of macrophages in joint *synovial tissue* of healthy subjects are poorly described. In RA, most knowledge of synovial myeloid cells is derived from studies of monocytes from RA *synovial fluid* that contribute to local production of inflammatory and joint-degrading mediators.^16–18^ However, monocytes are not present in the synovial fluid from healthy subjects^19^ and from patients with RA in remission.^20^

STMs are the most common resident immune cells in the healthy synovial membrane,^21^ and dendritic cells and lymphocytes are scarce. In active RA with myeloid and lymphoid synovitis, the synovial membrane is leucocyte-rich, including an increased number of proinflammatory macrophages that likely differentiate locally from blood monocytes attracted to synovial tissue and fluid by local chemokines,^22^ and these macrophages are the main producers of pathogenic TNF.^4 11 17^ In remission, most of the synovial inflammation resolves, but STMs in the lining layer persist.^11^ It would be important to establish if these macrophages have the same phenotype as those in healthy synovium. The constitutive presence of tissue macrophages in healthy synovial tissue and the sustained presence in patients with RA during remission suggests that these subpopulations of STMs may have a role in synovial homeostasis.^11^

## The concept of tissue-resident and monocyte-derived macrophages

Mouse models show that most healthy organs contain tissue-resident macrophages that originate from prenatal precursors and are essential for maintaining immune homeostasis and intact organ function. In response to inflammation, a different tissue macrophage population differentiates from monocytes that migrate transiently from the circulation. The relative roles of these macrophage populations determine chronicity or resolution of inflammation.^23^

**Figure 1:**
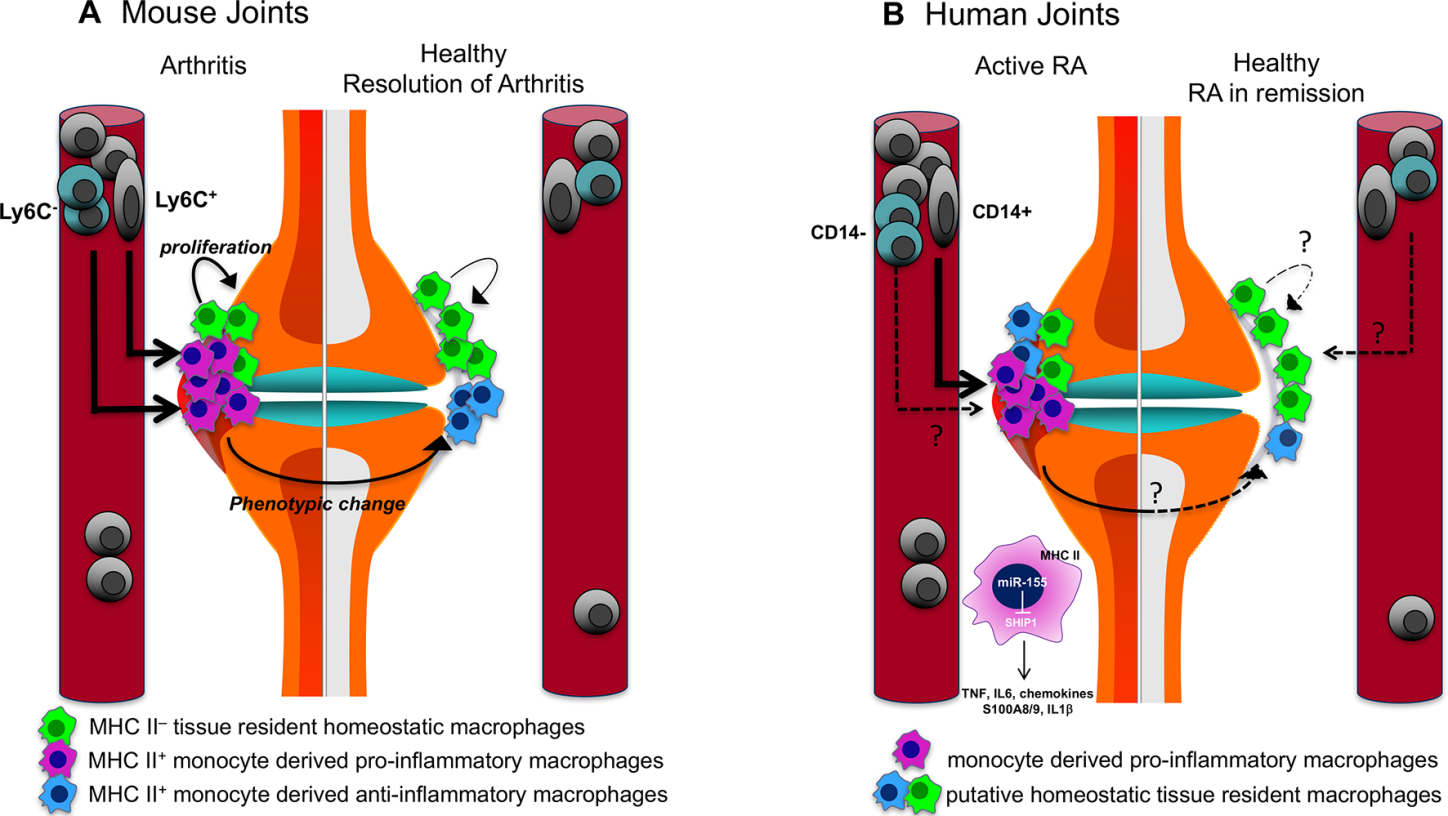
The role of synovial tissue macrophages in arthritis. (A) The mouse synovium contains major histocompatibility complex (MHC) class II negative (MHC-II^−^) tissue-resident macrophages that originate from prenatal precursors and have the capacity to proliferate. On induction of experimental arthritis, there is an influx of monocytes (Ly6C**^+^**or Ly6C**^−^**, depending of the nature of the induction) that differentiate into MHC class II positive (**^+^**) proinflammatory macrophages that mediate joint pathology. Resolution of arthritis is associated with a phenotypic change of the monocyte-derived macrophages from proinflammatory to anti-inflammatory, along with the contribution of tissue-resident macrophages. (B) In humans, the synovial membrane of healthy subjects and of patients with RA in sustained remission contain macrophages in the lining layer. Their origin and function are unknown. In patients with active arthritis, the CD14^+^ subpopulation of infiltrating monocytes contributes to the increased MHC-II^+^ proinflammatory macrophage pool in the inflamed synovium. Proinflammatory macrophages of patients with arthritis produce a broad range of inflammatory mediators, and their phenotype is maintained by the miR-155/SHIP-1 pathway. PI3K, phosphatidylinositol-4,5-bisphosphate 3-kinase; RA, rheumatoid arthritis; SHIP-1, phosphatidylinositol-3,4,5-trisphosphate 5-phosphatase 1; TLR, Toll-like receptors.

The ontogeny and precise role of human STMs in joint homeostasis are unknown. Animal studies suggest that mouse resident STMs are of prenatal origin and can proliferate locally in the tissue, whereas synovial tissue proinflammatory macrophages differentiate from either of two subpopulations of blood monocytes (Ly6C**^pos^** and Ly6C**^neg^**) that traffic into the inflamed synovium dependent on the nature of the trigger of the joint inflammation.^15 24^ The Ly6C**^neg^** patrolling monocytes differentiate into proinflammatory macrophages that mediated joint pathology in an antibody transfer model of arthritis, while Ly6C**^pos^** classical monocytes gave rise to synovial macrophages that drive inflammation in adjuvant-induced and antigen-induced arthritis.^24^ It is likely that the difference in chemokines induced by immune complexes or adjuvant in these models were responsible for attracting different populations of monocytes into the joints. In vivo tracking of labelled blood monocytes (CD14^+^ population that resembles mouse Ly6C^+^) in patients with RA has confirmed their migration into the inflamed synovium where they add to the tissue macrophage pool during inflammation.^25 26^ The traffic of other monocyte subpopulations into and within synovial tissue remains to be confirmed (figure 1).

The distinct functions of mouse tissue-resident and monocyte-derived macrophage populations in animal models of disease have improved our understanding of organ homeostasis.^27^ Tissue-resident macrophages can be self-renewing, controlled by the transcription factor Gata6, and maintain tissue immune homeostasis,^28^ whereas monocyte-derived macrophages predominate and drive inflammation in chronic disease models.^15 29^ Successful resolution of inflammation and reinstatement of tissue homeostasis requires differentiation of these proinflammatory macrophages towards an anti-inflammatory phenotype, and the activation/expansion of resident tissue macrophages^15 29 30^ that synergise for tissue homeostasis by production of anti-inflammatory/immune regulatory mediators (eg, resolvins, IL-10 and transforming growth factor beta (TGFβ)).^31–33^ In addition, tissue-resident macrophages contribute to tissue remodelling by removing apoptotic cells (efferocytosis), metabolic products and damaged tissue components^34–37^ and by reinstating tissue spatial organisation, for example, pigment cell distribution in zebrafish skin.^38^ In addition to the shared immune regulatory functions, mouse tissue-resident macrophages show tissue-specific homeostatic properties that are determined by the physiological needs of their specific environment. Tissue-resident macrophage differentiation and function is governed by tissue-specific cues and ‘on demand’ signals. Examples of these have been shown in an elegant study by van der Laar and colleagues in which either yolk-sac macrophages, fetal liver macrophages or bone marrow monocytes, on transfer into empty lung alveolar niches, indistinguishably differentiated into alveolar macrophages with the characteristic local functional regulation of lung surfactants.^39^ Transfer of mature macrophages from other tissues could not replicate this differentiation, thus confirming that the plasticity of macrophage maturation is optimal at the precursor stage and that tissue-specific cues determine local macrophage specialisation.

**Figure 2:**
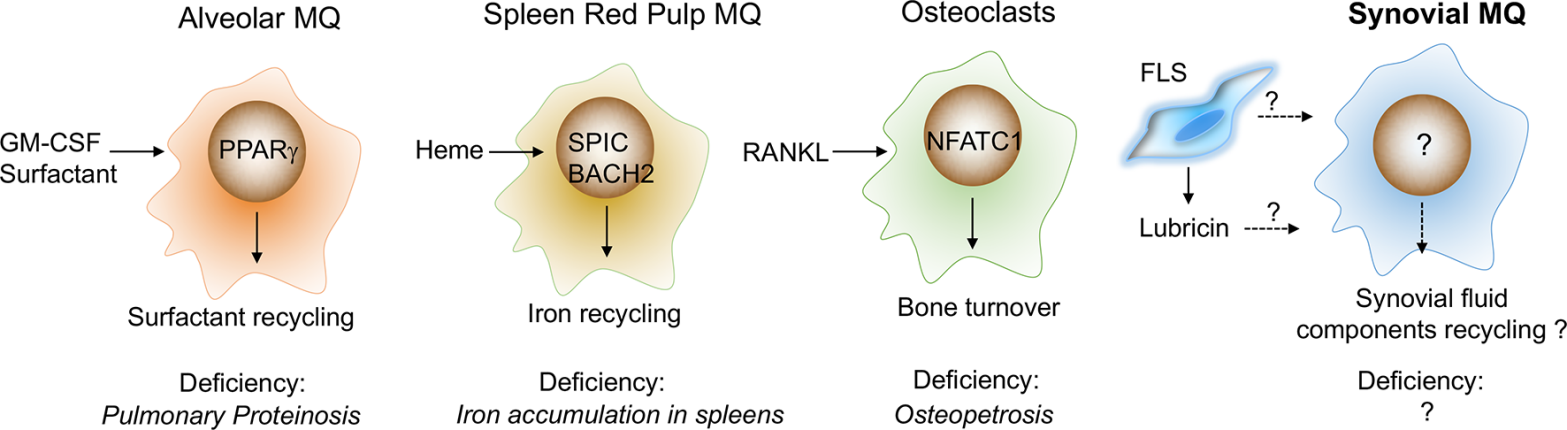
What is the tissue-specific function of synovial-resident macrophages? Tissue-specific functions of resident-tissue macrophages are induced by local cues. Their function in the synovium is unknown, but we can speculate by reference to other tissues. In the lung, for example, the differentiation of alveolar macrophages is driven by lung epithelial cell-derived GM-CSF, and recycling of surfactant is regulated by transcription factor PPARγ. Deficiency of alveolar macrophages, GM-CSF or PPARγ leads to pulmonary proteinosis. Spleen red pulp macrophages specialised in iron-recycling. This function is induced by heme derived from erythrocyte degradation and executed by BACH2 and SPIC. Selective deficiency of red pulp macrophages (deficiency of SPIC) leads to aberrant iron metabolism. Osteoclasts are bone macrophages that specialise in bone degradation. Their functional programme is induced by RANKL and executed by multiple transcription factors, including NFATC1. Deficiency in functional osteoclasts (RANKL**^−/^**^−^**** or M-CSF**^-**^−^**/**^−^**^**) leads to an increase in bone mass (osteopetrosis). Speculatively, in healthy joints, synovial tissue macrophages may specialise in recycling lubricin, the lubricating components of synovial fluid and in providing regulatory factors for cartilage and bone turnover. BACH2, transcription regulator protein BACH2; FLS, fibroblast-like synoviocytes; GM-CSF, granulocyte macrophage colony-stimulating factor; M-CSF, macrophage colony-stimulating factor; MQ, macrophages; NFATC1, nuclear factor of activated T cells, cytoplasmic 1;  PPARγ, peroxisome proliferator-activated receptor gamma; RANKL, receptor activator of nuclear factor kappa-B ligand; SPIC, transcription factor Spi-C.

These tissue-specific cues include a combination of cell contact and soluble factors.^40^ These have not been delineated in the synovium, but equivalent examples have been described in other tissues (figure 2), for example, direct contact with neurons is required for macrophage precursors to develop into microglia.^41^ The tissue-soluble factors include cytokines and tissue breakdown products. The differentiation of alveolar macrophages requires lung epithelial cell-derived granulocyte macrophage colony-stimulating factor (GM-CSF) and the induction of the transcription factor peroxisome proliferator-activated receptor gamma (PPARγ) that contributes to tissue-specific local function by regulating lung surfactants.^42^ The local differentiation of spleen red pulp macrophages requires the transcription factor BACH1 that recognises heme from erythrocyte degradation and induces their specialised iron-recycling function.^43^ Retinoic acid produced by the omentum is key in the differentiation of peritoneal macrophages that function to control IgA production by B-1 cells,^44^ and receptor activator of nuclear factor kappa-B ligand (RANKL) expressed in bone osteoblasts drives the differentiation of macrophages (osteoclasts) with bone-resorbing function.^45^ A combination of these tissue-specific cues and transient ‘on-demand’ signals, (eg, tissue injury creates signals to remove apoptotic inflammatory cells)^32 37^ induces programmes that are common for all tissue macrophages or programmes that are specific for a particular tissue. These programmes enable cells to perform general immune homeostatic functions, for example, Mer tyrosine protein kinase receptor (MerTK)-mediated efferocytosis and production of anti-inflammatory resolvins,^32 37^ or tissue-specific functions, for example, iron recycling by the spleen macrophages.^46^ These tissue-specific transcriptomic programmes are coordinated by specific transcription factors, for example, *SPIC* 46 in spleen red pulp, *LXRα* 47 in spleen marginal zone, *PPARγ* 42 and *Bach2* 48 in alveolar macrophages, *GATA6* 34 in peritoneal macrophages and *NR4A1* 49 in thymic macrophages. These cooperate with macrophage lineage master regulator transcription factors such as *PU.1* induced by macrophage colony-stimulating factor (M-CSF)/IL-34 that maintain universal macrophage-specific gene expression.^50^ Alveolar macrophage deficiency in humans and mice results in pulmonary alveolar proteinosis caused by the progressive accumulation of surfactant,^39 51^ and mice deficient in functional spleen red pulp macrophages develop a disorder of iron homeostasis.^46^ These examples illustrate the critical role of tissue-resident macrophages in the maintenance of tissue homeostasis (figure 2).

## Human homeostatic STMs: friend

The diversity of human STMs in health and disease is poorly described, while their tissue-specific functions are unknown. In healthy joints, STMs are located on the surface of the synovial membrane and are in contact with synovial fluid, suggesting that they may specialise in recycling the lubricating components of synovial fluid (analogous to alveolar macrophage recycling surfactant) and in providing regulatory factors for cartilage and bone turnover. Similar to other tissue-resident macrophages, they may also remove cell debris and pathogens to prevent sterile and septic inflammation. We review here the evidence for the potential homeostatic role of human synovial tissue-resident macrophages. Most of these data are historical and/or based on immunohistochemistry and is therefore of limited functional and molecular resolution. Early studies described three subtypes of STMs distinguishable by staining with 25F9, 27E10 and RM3/1 antibody clones. Their tissue distribution differed depending on whether the synovial tissue was normal, inflamed or resolving in studies on septic arthritis^52^ suggesting that they had distinct functions. In patients with osteoarthritis the 25F9**^+^** macrophages predominated in the non-inflammatory synovium^53^ and in the non-inflamed areas in the synovium of septic arthritis,^52^ whereas macrophages in the inflamed areas in the synovium of septic arthritis were 27E10^+^.^52^ The latter also dominated in the synovial tissues of patients with active RA (more information in the next section).^54^ 25F9**^+^** macrophages abundantly expressed IL-1R antagonist,^53^ suggesting an anti-inflammatory function. In healthy joints, most STMs contained phagosomes and were RM3/1**^+^** (now known to recognise the scavenger receptor CD163), suggesting that they are strongly phagocytic.^3 55^ They also expressed major histocompatibility complex (MHC)-II, IL-1R antagonist and the inhibitor of bone degradation, osteoprotegerin (OPG),^3 21 56^ and they were negative for proinflammatory cytokines (eg, TNF and IL1β) and the bone resorption cytokine RANKL,^56^ suggesting a joint protective function against inflammation and damage. Furthermore, in contrast to inflamed joints, these macrophages colocalise with mesenchymal progenitor cells^57^ suggesting a role in regulating repair.

These observations indicate the presence of subsets of tissue macrophages with functional characteristics suggesting a potential homeostatic role controlling inflammation, maintaining tissue integrity and bone protection, thus preserving healthy joint structure and function.^11^ Experimental models of arthritis support this hypothesis. Healthy mouse synovium contains a tissue-resident macrophage population that has a gene signature distinct from monocyte-derived macrophages. This includes expression of inhibitors of inflammation, SHIP-1 and MerTK, and these macrophages were crucial in preventing inflammatory responses in mouse synovium and in resolution of arthritis in the antibody transfer arthritis model.^15 24^ These data support the concept that joint immune homeostasis could be reinstated by distinct STM subpopulation.

## Human inflammatory STMs: foe

In active RA, fuelled by an influx of blood monocytes,^26^ the total number of STMs increased in all types of synovitis,^9^ particularly in the sublining layer of myeloid and lymphoid types. They are predominantly proinflammatory and produce CXCL4 and CXCL7 (chemokines recruiting neutrophils and blood monocytes) particularly in early RA,^22^ and TNF^58^ and other pro-inflammatory cytokines (IL-15,^59^ IL-1β,^53^ IL-6, GM-CSF and TGFβ^60^) throughout disease progression. These macrophages are 27E10**^+^**, which recognises an epitope on the alarmins S100A8/9.^61^ These S100A8/9**^+^** macrophages are susceptible to anti-TNF therapy^54^ after which they are removed by rapid efflux.^26^ RM3/1 (CD163) macrophages, typically found in healthy joints, are also present in RA synovium. They were found to be localised far from IFNγ positive T cells,^62^ and their numbers were unaffected by anti-TNF therapy^54^ suggesting that they have distinct functions. Historical ex vivo functional studies demonstrated that total macrophages isolated from active RA synovial tissue spontaneously released proinflammatory mediators (eg, TNF, IL-1β, IL-6,^63^ CXCL8^64^ and CCL2^65^) and are able to stimulate autologous T cells.^66^ Staining of these macrophages with the antibody recognising CD13, expressed on bone marrow-derived myeloid cells, alluded to the presence of at least two functionally distinct subpopulations that differed in their angiogenic properties. Only macrophages that were CD13 positive promoted angiogenesis, implying a role in vascularisation and hyperplasia of the RA synovium.^67 68^

**Figure 3:**
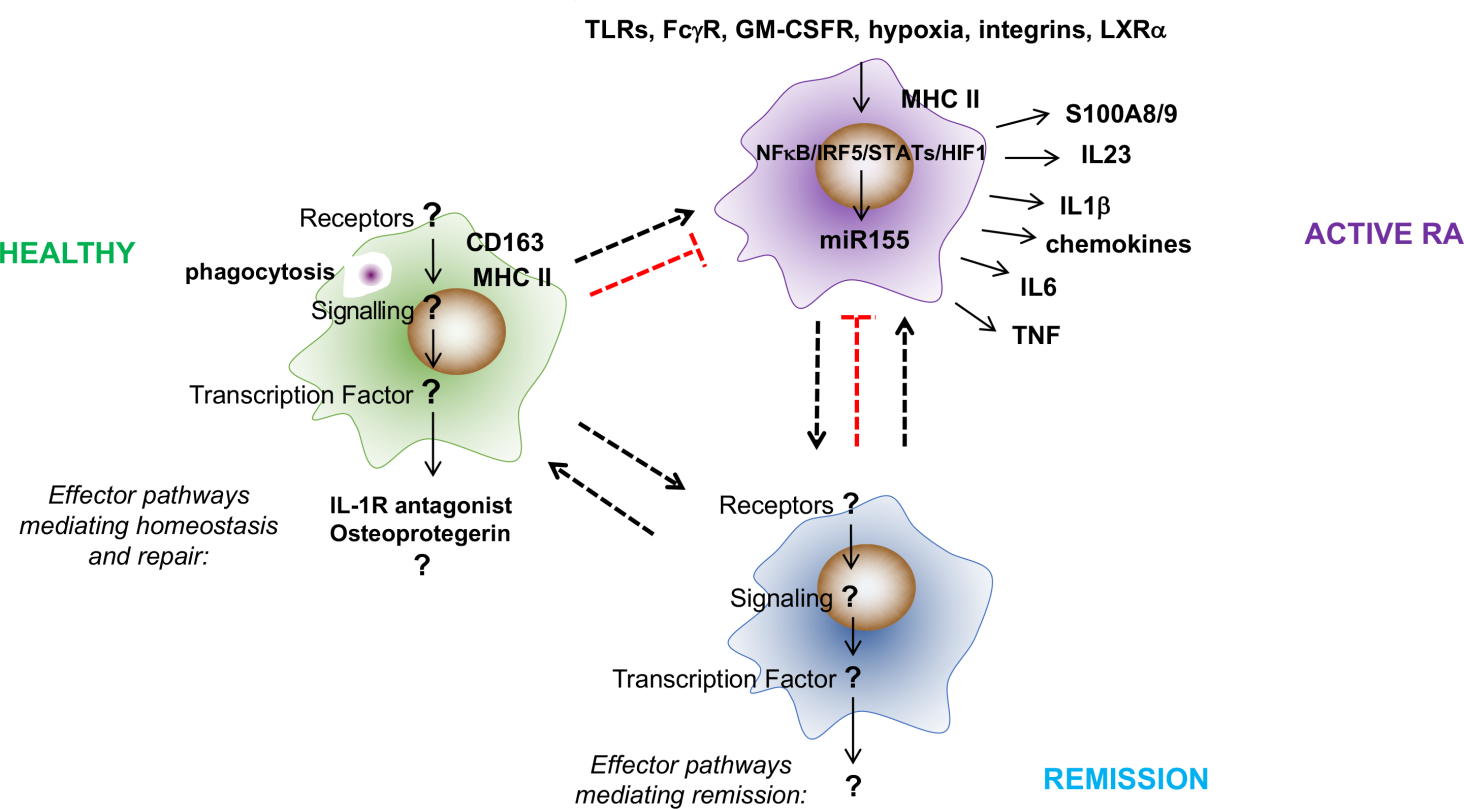
What are the effector pathways, transcriptional regulators and activators of synovial tissue macrophages in healthy donors and in patients with RA in remission? The constitutive presence of tissue macrophages in healthy synovial tissue and the sustained presence of some synovial tissue macrophages in patients with RA during remission suggests that these subpopulations have a role in maintaining and reinstating synovial homeostasis. However, little is known about the effector pathways and stimuli and transcription factors that execute their function. Synovial tissue macrophages in healthy subjects express the scavenger receptor CD163, suggesting that they are strongly phagocytic. They also express MHC-II, IL-1R-antagonist and the inhibitor of bone degradation, osteoprotegerin (OPG), and they are negative for proinflammatory cytokines (eg, TNF and IL1β) and the bone resorption cytokine RANKL, suggesting a joint protective function against inflammation and damage. In contrast, the phenotype of proinflammatory synovial tissue macrophages is well described. These cells are MHC-II positive and produce a broad range of inflammatory mediators (eg, TNF, IL1β, IL-6, IL-23 and S100A8/9) that drive local and systemic pathologies in RA. Their activation is sustained by a variety of local stimuli that include endogenous TLR ligands, immune complexes, oxidised lipids, hypoxia and integrin-mediated contact with synovial fibroblasts and T cells. This proinflammatory programme is executed by NFκB, IRF5, STAT1/5 and HIF1α and is maintained by microRNA-155. FcγR, Fc gamma receptor; GM-CSFR, granulocyte macrophage colony-stimulating factor receptor; IL, interleukin; IRF, interferon regulatory factor; LXRα, liver X receptor alpha; NFκB, nuclear factor kappa-light-chain-enhancer of activated B cells; RA, rheumatoid arthritis; STAT, signal transducer and activator of transcription; TLR, Toll-like receptors; TNF, tumour necrosis factor.

The activation of proinflammatory macrophages in humans and mice is governed by the transcription factors NFκB, IRF5 and STAT1/5^69 70^ and is maintained by microRNA-155. We showed that increased miR-155 prevented the translation of its mRNA epigenetic target SHIP-1, and thereby removed its negative feedback control of inflammatory TLR and PI3K signalling, thus permitting sustained production of cytokines and chemokines by these macrophages.^17 71^ Most RA STMs express high levels of miR-155 and commensurately low levels of SHIP-1, which is in sharp contrast to macrophages from non-inflamed synovium where the majority lack miR-155 and have high expression of SHIP-1^17^ (figure 3).

Together these data provide evidence for *different macrophage subpopulations in human synovium with potentially homeostatic or inflammatory properties.* However, the prospect of therapeutic translation of the homeostatic potential of STM subpopulations requires more precise understanding of their phenotypic heterogeneity and function. The rapid progress in using synovial tissue biopsy to study disease pathogenesis^72^ and the current advances in a single cell sequencing^73^ could help to reveal their biology.

## Potential tissue-specific ‘cues’ and ‘on demand’ determinants of synovial tissue macrophage homeostatic and inflammatory functions

Okabe and Medzhitov proposed a model in which the tissue-resident macrophage phenotype is determined by core transcriptomic modules induced, for example, by M-CSF/PU.1 that are common for all macrophages. Thereafter, tissue-specific transcriptomic modules are induced by tissue ‘cues’ (eg, GM-CSF in alveolar macrophages) that determine tissue-specific function (eg, recycling of surfactant in the lung) to maintain organ-specific homeostasis. Changes in the local environment, for example, infection or tissue injury, induce ‘on demand’ modules to eliminate the stressor, for example, chemokine production, to initiate the recruitment of neutrophils and monocytes that become a source of inflammatory macrophages^74^ or efferocytosis^37^ to facilitate the reinstatement of tissue homeostasis at the later stages of inflammation.^32 40^

The tissue-specific cues determining the phenotype and transcriptomic programme of synovial tissue-resident macrophages in health and those present in patients with RA in remission are unknown. Local differentiation of microglia requires direct contact of primitive macrophage precursors with neurons,^41^ therefore by analogy, the interaction of macrophage precursors with lining synovial fibroblasts that undergo biomechanical stretch may trigger their local differentiation into synovial tissue-resident macrophages. The characteristic ECM produced by synovial fibroblasts (eg, lubricin and hyaluronic acids), by analogy to the profound impact of ECM produced by lung fibroblasts on the transcriptome of recruited myeloid cells,^75 76^ could affect macrophage differentiation and survival by binding to macrophage CD44^76^ or TLR2.^77^ Uncovering the transcriptomic programme, molecular regulators and cues that drive synovial tissue-resident macrophage function will aid the understanding of mechanisms of joint homeostasis and provide novel therapeutic approaches to reinstate joint immune homeostasis in patients with arthritis.

**Figure 4:**
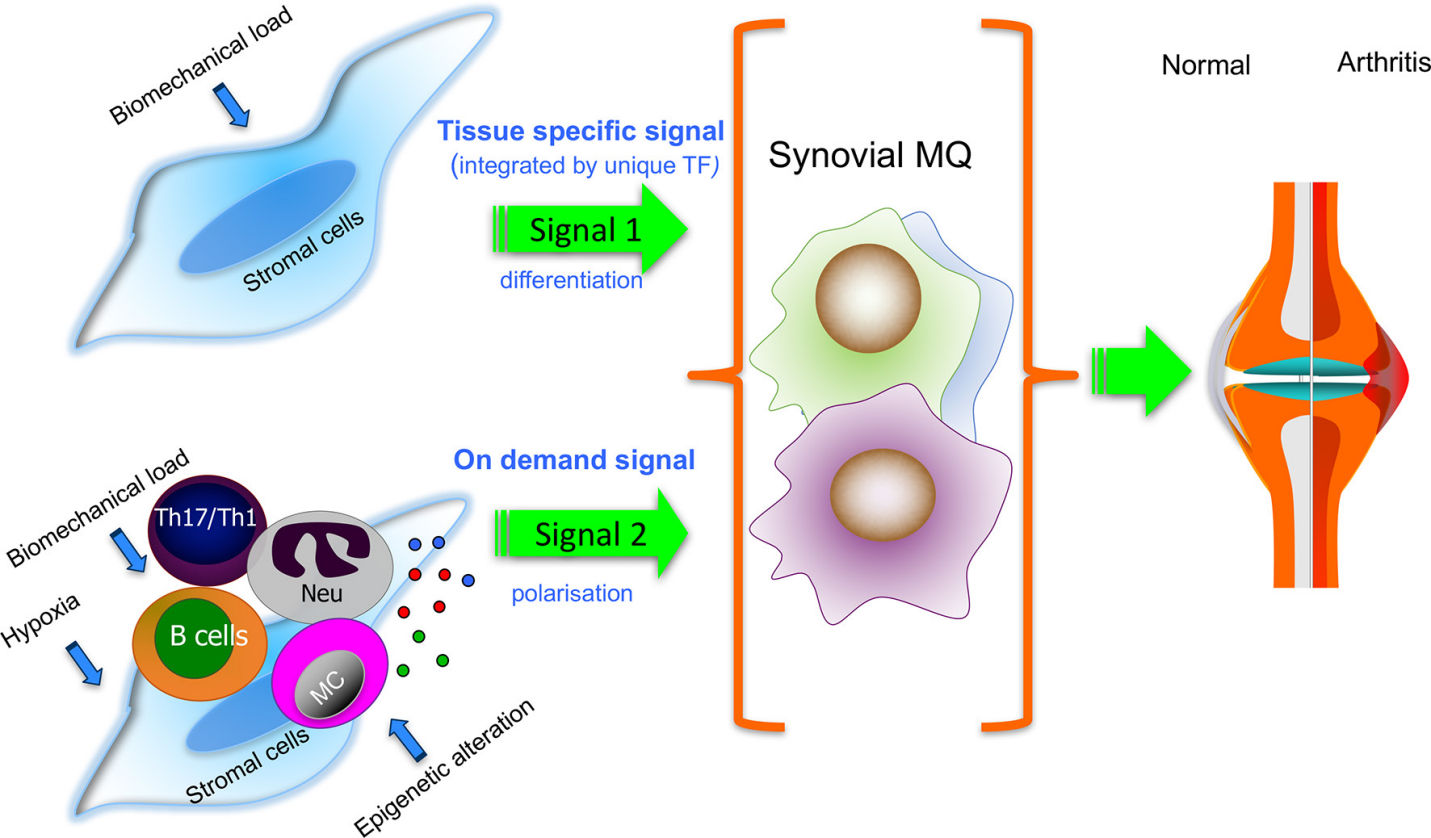
Putative contribution of tissue-specific (signal 1) and the local polarisation environment (signal 2) to the phenotype of synovial tissue macrophages in homeostasis and inflammation. Tissue-specific cues (signal 1), for example, from stretched synovial fibroblasts (FLS) to macrophage precursors may induce the synovial tissue macrophage programme that maintains synovial homeostasis. In the inflamed RA synovium, tissue-specific cues are modulated by proinflammatory on-demand signals (signal 2; eg, provided by epigenetically changed FLS, leucocytes and hypoxia). Most understanding is based on experimental models, and it is unknown whether distinct human synovial tissue macrophage subpopulations would respond differently to the synovial tissue-specific and ‘on-demand’ proinflammatory cues. Dissecting these pathways will improve our understanding of the mechanisms of successful versus failed joint homeostasis. MC, mast cells; MQ, macrophages; Neu, neutrophils; TF, transcription factor; Th17/Th1, T helper 1 and 17.

In the tissue environment of the RA synovium, tissue-specific cues are modulated by tissue injury and proinflammatory ‘on-demand’ signals, which mobilise monocytes and instruct their differentiation to monocyte-derived pro-inflammatory macrophages.^4 71 78 79^ It is unknown whether distinct human tissue-resident and monocyte-derived synovial tissue macrophage subpopulations would respond differently to the synovial tissue-specific and ‘on demand’ proinflammatory cues (figure 4). In mouse tissues, recruitment of blood monocytes in response to ‘on-demand’ signal is often concomitant with a loss of tissue-resident macrophages due to emigration or death.^80^ In the synovium of active patients with RA, both S100A8/9**^+^**and CD163**^+^**macrophages are present, although the latter in much lower numbers.^54^ Similarly, in mouse models of arthritis, tissue-resident macrophages were present at the peak of inflammation, along with monocyte-derived macrophages, although outnumbered and with distinct transcriptomic profiles. For example, monocyte-derived macrophages expressed high levels of proinflammatory mediators, for example, IL-1β, IL-12, CD80 and CD86, compared with resident macrophages, while the latter upregulated efferocytosis receptors.^15^ This suggests that, at least in the mouse, tissue-resident and monocyte-derived macrophages may respond differently to ‘on demand’ signals. In RA synovium, the phenotype of monocyte-derived macrophages is shaped by the interaction with synovial fibroblasts that are epigenetically reprogrammed to produce a wide range of mediators,^81^ for example, GM-CSF that potentiates the proinflammatory activation of macrophages.^82 83^ In addition, danger-associated molecular pattern molecules (DAMPs) released in the damage joint, for example, tenascin-C, bind to TLR4 and trigger proinflammatory cytokine production by macrophages.^84^ ACPA produced locally in lymphoid type of synovitis forms immune complexes with citrullinated fibrinogen and induces TNF production via synergistic binding to FcγR and TLR4.^85^ Oxysterols, enriched in RA synovial fluid, bind to LXRα in synovial macrophages and enhance DAMPs-induced TNF, IL-6 and IL-1β production.^86^ Memory T cells, recruited and expanded in synovial fluid by cytokines (eg, IL-15, IL-6 and TNF), trigger substantial production of TNF by macrophages on direct integrin-mediated contact,^87 88^ while IFNγ released by RA citrullinated peptide autoreactive T cells^89^ enhances the ability of macrophages to present antigen and thereby their potential to activate autoreactive memory T cells. The hypertrophic synovium creates a hypoxic environment,^90^ and recruited macrophages adapt by changing their metabolism and phenotype. This response, mediated by hypoxia inducible transcription factor 1 (HIF-1α), leads to an increased production of proinflammatory cytokines and can redirect differentiation towards bone-resorbing osteoclasts.^91^ For example, HIF-1α abrogates the mTORC1/IL-10-induced negative feedback mechanism of macrophage activation^92^ while stimulating IL-1β production.^93^ In addition, hypoxia decreases the expression of COMMD1 (an inhibitor of macrophage to osteoclast differentiation) and can thereby facilitate synovial macrophage-driven pathogenic bone resorption.^91^

Many of these signals increase the expression of miR-155, which locks the macrophages in a proinflammatory state.^17 94^ In mouse models of arthritis, at some time-point, there is a phenotypic change of monocyte-derived macrophages from proinflammatory to anti-inflammatory, which is induced by an as-yet unidentified signal. This phenotypic change in monocyte-derived macrophages and the functions of tissue-resident macrophages are required for the resolution of arthritis.^15^

In RA, the role of recruited synovial macrophages in mediating remission of disease is uncertain. The observation that alleviation of disease with anti-TNF therapy leads to a decrease of joint-infiltrating S100A8/9**^+^** macrophages,^26^ presumably by enhanced efflux of these cells from the synovium,^26^ while the number of potential resident-tissue (CD163^+^) macrophages remained unchanged^54^ would argue against an active role of monocyte-derived macrophages in the resolution of RA.

## Summary

There is evidence for the presence of different subpopulations of synovial tissue macrophages with potentially distinct homeostatic versus inflammatory functions. While synovial macrophage inflammatory pathways are relatively well described and therapeutically targeted, little is known about the synovial tissue macrophages constitutively present in healthy subjects and persistent in patients with RA during sustained remission. Understanding the biology of these cells could help to reveal the mechanisms of synovial immune homeostasis and could offer the prospect of novel strategies for treatment of joint diseases.

## References

[1] Firestein GS, Gabriel SE, McInnes IB, et al Kelley and Firestein’s textbook of rheumatology. 10th edn Amsterdam, Netherlands: Elsevier, 2016.

[2] Alivernini S, Peluso G, Fedele AL, et al Tapering and discontinuation of TNF-α blockers without disease relapse using ultrasonography as a tool to identify patients with rheumatoid arthritis in clinical and histological remission. Arthritis Res Ther 2016;18:39 10.1186/s13075-016-0927-z 26842890 PMC4741059

[3] Singh JA, Arayssi T, Duray P, et al Immunohistochemistry of normal human knee synovium: a quantitative study. Ann Rheum Dis 2004;63:785–90. 10.1136/ard.2003.013383 15194572 PMC1755068

[4] Firestein GS, McInnes IB Immunopathogenesis of rheumatoid arthritis. Immunity 2017;46:183–96. 10.1016/j.immuni.2017.02.006 28228278 PMC5385708

[5] Arthritis Research UK charity. Arthritis impacts the lives of over 10 million adults in the UK. 2017 http://www.arthritisresearchuk.org/

[6] Okada Y, Wu D, Trynka G, et al Genetics of rheumatoid arthritis contributes to biology and drug discovery. Nature 2014;506:376–81. 10.1038/nature12873 24390342 PMC3944098

[7] Smolen JS, Aletaha D, McInnes IB Rheumatoid arthritis. Lancet 2016;388:2023–38. 10.1016/S0140-6736(16)30173-8 27156434

[8] Pitzalis C, Kelly S, Humby F New learnings on the pathophysiology of RA from synovial biopsies. Curr Opin Rheumatol 2013;25:334–44. 10.1097/BOR.0b013e32835fd8eb 23492740

[9] Dennis G, Holweg CT, Kummerfeld SK, et al Synovial phenotypes in rheumatoid arthritis correlate with response to biologic therapeutics. Arthritis Res Ther 2014;16:R90 10.1186/ar4555 25167216 PMC4060385

[10] Nagy G, van Vollenhoven RF Sustained biologic-free and drug-free remission in rheumatoid arthritis, where are we now? Arthritis Res Ther 2015;17:181 10.1186/s13075-015-0707-1 26235544 PMC4522973

[11] Alivernini S, Tolusso B, Petricca L, et al Synovial features of patients with rheumatoid arthritis and psoriatic arthritis in clinical and ultrasound remission differ under anti-TNF therapy: a clue to interpret different chances of relapse after clinical remission? Ann Rheum Dis 2017;76:1228–36. 10.1136/annrheumdis-2016-210424 28119289 PMC5530352

[12] van der Heijde D, Klareskog L, Boers M, et al Comparison of different definitions to classify remission and sustained remission: 1 year TEMPO results. Ann Rheum Dis 2005;64:1582–7. 10.1136/ard.2004.034371 15860509 PMC1755254

[13] Kawashiri SY, Fujikawa K, Nishino A, et al Ultrasound-detected bone erosion is a relapse risk factor after discontinuation of biologic disease-modifying antirheumatic drugs in patients with rheumatoid arthritis whose ultrasound power Doppler synovitis activity and clinical disease activity are well controlled. Arthritis Res Ther 2017;19:108 10.1186/s13075-017-1320-2 28545509 PMC5445491

[14] Dirven L, Güler-Yüksel M, de Beus WM, et al Changes in hand bone mineral density and the association with the level of disease activity in patients with rheumatoid arthritis: bone mineral density measurements in a multicenter randomized clinical trial. Arthritis Care Res 2011;63:1691–9. 10.1002/acr.20612 21905248

[15] Misharin AV, Cuda CM, Saber R, et al Nonclassical Ly6C(-) monocytes drive the development of inflammatory arthritis in mice. Cell Rep 2014;9:591–604. 10.1016/j.celrep.2014.09.032 25373902 PMC4223808

[16] Rajasekhar M, Olsson AM, Steel KJ, et al MicroRNA-155 contributes to enhanced resistance to apoptosis in monocytes from patients with rheumatoid arthritis. J Autoimmun 2017;79:53–62. 10.1016/j.jaut.2017.01.002 28118944 PMC5397583

[17] Kurowska-Stolarska M, Alivernini S, Ballantine LE, et al MicroRNA-155 as a proinflammatory regulator in clinical and experimental arthritis. Proc Natl Acad Sci U S A 2011;108:11193–8. 10.1073/pnas.1019536108 21690378 PMC3131377

[18] Evans HG, Gullick NJ, Kelly S, et al In vivo activated monocytes from the site of inflammation in humans specifically promote Th17 responses. Proc Natl Acad Sci U S A 2009;106:6232–7. 10.1073/pnas.0808144106 19325128 PMC2669354

[19] Jepsen KJ Systems analysis of bone. Wiley Interdiscip Rev Syst Biol Med 2009;1:73–88. 10.1002/wsbm.15 20046860 PMC2790199

[20] Koch AE Chemokines and their receptors in rheumatoid arthritis: future targets? Arthritis Rheum 2005;52:710–21. 10.1002/art.20932 15751074

[21] Smith MD The normal synovium. Open Rheumatol J 2011;5:100–6. 10.2174/1874312901105010100 22279508 PMC3263506

[22] Yeo L, Adlard N, Biehl M, et al Expression of chemokines CXCL4 and CXCL7 by synovial macrophages defines an early stage of rheumatoid arthritis. Ann Rheum Dis 2016;75:763–71. 10.1136/annrheumdis-2014-206921 25858640 PMC4819606

[23] Ginhoux F, Guilliams M Tissue-resident macrophage ontogeny and homeostasis. Immunity 2016;44:439–49. 10.1016/j.immuni.2016.02.024 26982352

[24] Weiss M, Byrne AJ, Blazek K, et al IRF5 controls both acute and chronic inflammation. Proc Natl Acad Sci U S A 2015;112:11001–6. 10.1073/pnas.1506254112 26283380 PMC4568217

[25] Thurlings RM, Wijbrandts CA, Bennink RJ, et al Monocyte scintigraphy in rheumatoid arthritis: the dynamics of monocyte migration in immune-mediated inflammatory disease. PLoS One 2009;4:e7865 10.1371/journal.pone.0007865 19924229 PMC2773010

[26] Herenius MM, Thurlings RM, Wijbrandts CA, et al Monocyte migration to the synovium in rheumatoid arthritis patients treated with adalimumab. Ann Rheum Dis 2011;70:1160–2. 10.1136/ard.2010.141549 21345816 PMC3086080

[27] Davies LC, Jenkins SJ, Allen JE, et al Tissue-resident macrophages. Nat Immunol 2013;14:986–95. 10.1038/ni.2705 24048120 PMC4045180

[28] Rosas M, Davies LC, Giles PJ, et al The transcription factor Gata6 links tissue macrophage phenotype and proliferative renewal. Science 2014;344:645–8. 10.1126/science.1251414 24762537 PMC4185421

[29] Davies LC, Rosas M, Jenkins SJ, et al Distinct bone marrow-derived and tissue-resident macrophage lineages proliferate at key stages during inflammation. Nat Commun 2013;4:1886 10.1038/ncomms2877 23695680 PMC3842019

[30] Huang QQ, Birket R, Doyle RE, et al Increased F4/80hi macrophages is associated with suppression of serum transfer induced arthritis in mice with flip reduced in myeloid cells. Rheumatoid Arthritis 2017 doi: 10.1002/art.40151[Epub ahead of print 17 May 2017]. 10.1002/art.40151 PMC5575965 28511285

[31] Bosurgi L, Bernink JH, Delgado Cuevas V, et al Paradoxical role of the proto-oncogene Axl and Mer receptor tyrosine kinases in colon cancer. Proc Natl Acad Sci U S A 2013;110:13091–6. 10.1073/pnas.1302507110 23878224 PMC3740859

[32] Triantafyllou E, Pop OT, Possamai LA, et al MerTK expressing hepatic macrophages promote the resolution of inflammation in acute liver failure. Gut 2017 doi: 10.1136/gutjnl-2016-313615[Epub ahead of print 30 Apr 2017]. 10.1136/gutjnl-2016-313615 PMC5868289 28450389

[33] Cai B, Thorp EB, Doran AC, et al MerTK cleavage limits proresolving mediator biosynthesis and exacerbates tissue inflammation. Proc Natl Acad Sci U S A 2016;113:6526–31. 10.1073/pnas.1524292113 27199481 PMC4988577

[34] Gautier EL, Shay T, Miller J, et al Gene-expression profiles and transcriptional regulatory pathways that underlie the identity and diversity of mouse tissue macrophages. Nat Immunol 2012;13:1118–28. 10.1038/ni.2419 23023392 PMC3558276

[35] Varol C, Mildner A, Jung S Macrophages: development and tissue specialization. Annu Rev Immunol 2015;33:643–75. 10.1146/annurev-immunol-032414-112220 25861979

[36] Nimmerjahn A, Kirchhoff F, Helmchen F Resting microglial cells are highly dynamic surveillants of brain parenchyma in vivo. Science 2005;308:1314–8. 10.1126/science.1110647 15831717

[37] Bosurgi L, Cao YG, Cabeza-Cabrerizo M, et al Macrophage function in tissue repair and remodeling requires IL-4 or IL-13 with apoptotic cells. Science 2017;356:1072–6. 10.1126/science.aai8132 28495875 PMC5556699

[38] Eom DS, Parichy DM A macrophage relay for long-distance signaling during postembryonic tissue remodeling. Science 2017;355:1317–20. 10.1126/science.aal2745 28209639 PMC5836293

[39] van de Laar L, Saelens W, De Prijck S, et al Yolk sac macrophages, fetal liver, and adult monocytes can colonize an empty niche and develop into functional tissue-resident macrophages. Immunity 2016;44:755–68. 10.1016/j.immuni.2016.02.017 26992565

[40] Okabe Y, Medzhitov R Tissue biology perspective on macrophages. Nat Immunol 2016;17:9–17. 10.1038/ni.3320 26681457

[41] Takata K, Kozaki T, Lee CZW, et al Induced-pluripotent-stem-cell-derived primitive macrophages provide a platform for modeling tissue-resident macrophage differentiation and function. Immunity 2017;47:183–98. 10.1016/j.immuni.2017.06.017 28723550

[42] Schneider C, Nobs SP, Kurrer M, et al Induction of the nuclear receptor PPAR-γ by the cytokine GM-CSF is critical for the differentiation of fetal monocytes into alveolar macrophages. Nat Immunol 2014;15:1026–37. 10.1038/ni.3005 25263125

[43] Haldar M, Kohyama M, So AY, et al Heme-mediated SPI-C induction promotes monocyte differentiation into iron-recycling macrophages. Cell 2014;156:1223–34. 10.1016/j.cell.2014.01.069 24630724 PMC4010949

[44] Okabe Y, Medzhitov R Tissue-specific signals control reversible program of localization and functional polarization of macrophages. Cell 2014;157:832–44. 10.1016/j.cell.2014.04.016 24792964 PMC4137874

[45] Lacey DL, Timms E, Tan HL, et al Osteoprotegerin ligand is a cytokine that regulates osteoclast differentiation and activation. Cell 1998;93:165–76. 10.1016/S0092-8674(00)81569-X 9568710

[46] Kohyama M, Ise W, Edelson BT, et al Role for Spi-C in the development of red pulp macrophages and splenic iron homeostasis. Nature 2009;457:318–21. 10.1038/nature07472 19037245 PMC2756102

[47] A-Gonzalez N, Guillen JA, Gallardo G, et al The nuclear receptor LXRα controls the functional specialization of splenic macrophages. Nat Immunol 2013;14:831–9. 10.1038/ni.2622 23770640 PMC3720686

[48] Nakamura A, Ebina-Shibuya R, Itoh-Nakadai A, et al Transcription repressor Bach2 is required for pulmonary surfactant homeostasis and alveolar macrophage function. J Exp Med 2013;210:2191–204. 10.1084/jem.20130028 24127487 PMC3804940

[49] Tacke R, Hilgendorf I, Garner H, et al The transcription factor NR4A1 is essential for the development of a novel macrophage subset in the thymus. Sci Rep 2015;5:10055 10.1038/srep10055 26091486 PMC4473761

[50] Mossadegh-Keller N, Sarrazin S, Kandalla PK, et al M-CSF instructs myeloid lineage fate in single haematopoietic stem cells. Nature 2013;497:239–43. 10.1038/nature12026 23575636 PMC3679883

[51] Suzuki T, Arumugam P, Sakagami T, et al Pulmonary macrophage transplantation therapy. Nature 2014;514:450–4. 10.1038/nature13807 25274301 PMC4236859

[52] Peters KM, Koberg K, Rosendahl T, et al Macrophage reactions in septic arthritis. Arch Orthop Trauma Surg 1996;115:347–50. 10.1007/BF00420330 8905111

[53] Cauli A, Yanni G, Panayi GS Interleukin-1, interleukin-1 receptor antagonist and macrophage populations in rheumatoid arthritis synovial membrane. Br J Rheumatol 1997;36:935–40. 10.1093/rheumatology/36.9.935 9376987

[54] De Rycke L, Baeten D, Foell D, et al Differential expression and response to anti-TNFalpha treatment of infiltrating versus resident tissue macrophage subsets in autoimmune arthritis. J Pathol 2005;206:17–27. 10.1002/path.1758 15809977

[55] Hogg N, Palmer DG, Revell PA Mononuclear phagocytes of normal and rheumatoid synovial membrane identified by monoclonal antibodies. Immunology 1985;56:673–81. 2416682 PMC1453794

[56] Smith MD, Barg E, Weedon H, et al Microarchitecture and protective mechanisms in synovial tissue from clinically and arthroscopically normal knee joints. Ann Rheum Dis 2003;62:303–7. 10.1136/ard.62.4.303 12634226 PMC1754505

[57] O’Brien K, Tailor P, Leonard C, et al Enumeration and localization of mesenchymal progenitor cells and macrophages in synovium from normal individuals and patients with pre-osteoarthritis or clinically diagnosed osteoarthritis. Int J Mol Sci 2017;18:774 10.3390/ijms18040774 PMC5412358 28379175

[58] Chu CQ, Field M, Feldmann M, et al Localization of tumor necrosis factor alpha in synovial tissues and at the cartilage-pannus junction in patients with rheumatoid arthritis. Arthritis Rheum 1991;34:1125–32. 10.1002/art.1780340908 1930331

[59] McInnes IB, al-Mughales J, Field M, et al The role of interleukin-15 in T-cell migration and activation in rheumatoid arthritis. Nat Med 1996;2:175–82. 10.1038/nm0296-175 8574962

[60] Chu CQ, Field M, Allard S, et al Detection of cytokines at the cartilage/pannus junction in patients with rheumatoid arthritis: implications for the role of cytokines in cartilage destruction and repair. Br J Rheumatol 1992;31:653–61. 10.1093/rheumatology/31.10.653 1393370

[61] Vogl T, Eisenblätter M, Völler T, et al Alarmin S100A8/S100A9 as a biomarker for molecular imaging of local inflammatory activity. Nat Commun 2014;5:4593 10.1038/ncomms5593 25098555 PMC4143994

[62] Fonseca JE, Edwards JC, Blades S, et al Macrophage subpopulations in rheumatoid synovium: reduced CD163 expression in CD4+ T lymphocyte-rich microenvironments. Arthritis Rheum 2002;46:1210–6. 10.1002/art.10207 12115225

[63] Okamoto H, Yamamura M, Morita Y, et al The synovial expression and serum levels of interleukin-6, interleukin-11, leukemia inhibitory factor, and oncostatin M in rheumatoid arthritis. Arthritis Rheum 1997;40:1096–105. 10.1002/art.1780400614 9182920

[64] Koch AE, Kunkel SL, Burrows JC, et al Synovial tissue macrophage as a source of the chemotactic cytokine IL-8. J Immunol 1991;147:2187–95. 1918955

[65] Koch AE, Kunkel SL, Harlow LA, et al Enhanced production of monocyte chemoattractant protein-1 in rheumatoid arthritis. J Clin Invest 1992;90:772–9. 10.1172/JCI115950 1522232 PMC329929

[66] Klareskog L, Forsum U, Kabelitz D, et al Immune functions of human synovial cells. Phenotypic and T cell regulatory properties of macrophage-like cells that express HLA-DR. Arthritis Rheum 1982;25:488–501. 10.1002/art.1780250502 6211176

[67] Koch AE, Polverini PJ, Leibovich SJ Stimulation of neovascularization by human rheumatoid synovial tissue macrophages. Arthritis Rheum 1986;29:471–9. 10.1002/art.1780290403 2423091

[68] Koch AE, Burrows JC, Skoutelis A, et al Monoclonal antibodies detect monocyte/macrophage activation and differentiation antigens and identify functionally distinct subpopulations of human rheumatoid synovial tissue macrophages. Am J Pathol 1991;138:165–73. 1987761 PMC1886034

[69] Udalova IA, Mantovani A, Feldmann M Macrophage heterogeneity in the context of rheumatoid arthritis. Nat Rev Rheumatol 2016;12:472–85. 10.1038/nrrheum.2016.91 27383913

[70] Lawrence T, Natoli G Transcriptional regulation of macrophage polarization: enabling diversity with identity. Nat Rev Immunol 2011;11:750–61. 10.1038/nri3088 22025054

[71] Elmesmari A, Fraser AR, Wood C, et al MicroRNA-155 regulates monocyte chemokine and chemokine receptor expression in Rheumatoid Arthritis. Rheumatology 2016;55:2056–65. 10.1093/rheumatology/kew272 27411480 PMC5088623

[72] Orr C, Vieira-Sousa E, Boyle DL, et al Synovial tissue research: a state-of-the-art review. Nat Rev Rheumatol 2017;13:463–75. 10.1038/nrrheum.2017.115 28701760

[73] Villani AC, Satija R, Reynolds G, et al Single-cell RNA-seq reveals new types of human blood dendritic cells, monocytes, and progenitors. Science 2017;356:eaah4573 10.1126/science.aah4573 28428369 PMC5775029

[74] Cailhier JF, Partolina M, Vuthoori S, et al Conditional macrophage ablation demonstrates that resident macrophages initiate acute peritoneal inflammation. J Immunol 2005;174:2336–42. 10.4049/jimmunol.174.4.2336 15699170

[75] Parker MW, Rossi D, Peterson M, et al Fibrotic extracellular matrix activates a profibrotic positive feedback loop. J Clin Invest 2014;124:1622–35. 10.1172/JCI71386 24590289 PMC3971953

[76] McKee CM, Penno MB, Cowman M, et al Hyaluronan (HA) fragments induce chemokine gene expression in alveolar macrophages. The role of HA size and CD44. J Clin Invest 1996;98:2403–13. 10.1172/JCI119054 8941660 PMC507693

[77] Alquraini A, Garguilo S, D’Souza G, et al The interaction of lubricin/proteoglycan 4 (PRG4) with toll-like receptors 2 and 4: an anti-inflammatory role of PRG4 in synovial fluid. Arthritis Res Ther 2015;17:353 10.1186/s13075-015-0877-x 26643105 PMC4672561

[78] Roberts CA, Dickinson AK, Taams LS The interplay between monocytes/macrophages and CD4(+) T cell subsets in rheumatoid arthritis. Front Immunol 2015;6:571 10.3389/fimmu.2015.00571 26635790 PMC4652039

[79] Stuhlmüller B, Ungethüm U, Scholze S, et al Identification of known and novel genes in activated monocytes from patients with rheumatoid arthritis. Arthritis Rheum 2000;43:775–90. 10.1002/1529-0131(200004)43:4<775::AID-ANR8>3.0.CO;2-7 10765922

[80] Barth MW, Hendrzak JA, Melnicoff MJ, et al Review of the macrophage disappearance reaction. J Leukoc Biol 1995;57:361–7. 7884305 10.1002/jlb.57.3.361

[81] Donlin LT, Jayatilleke A, Giannopoulou EG, et al Modulation of TNF-induced macrophage polarization by synovial fibroblasts. J Immunol 2014;193:2373–83. 10.4049/jimmunol.1400486 25057003 PMC4135020

[82] Achuthan A, Cook AD, Lee MC, et al Granulocyte macrophage colony-stimulating factor induces CCL17 production via IRF4 to mediate inflammation. J Clin Invest 2016;126:3453–66. 10.1172/JCI87828 27525438 PMC5004969

[83] Hamilton JA, Piccoli DS, Cebon J, et al Cytokine regulation of colony-stimulating factor (CSF) production in cultured human synovial fibroblasts. II. Similarities and differences in the control of interleukin-1 induction of granulocyte-macrophage CSF and granulocyte-CSF production. Blood 1992;79:1413–9. 1372187

[84] Midwood K, Sacre S, Piccinini AM, et al Tenascin-C is an endogenous activator of toll-like receptor 4 that is essential for maintaining inflammation in arthritic joint disease. Nat Med 2009;15:774–80. 10.1038/nm.1987 19561617

[85] Sokolove J, Zhao X, Chandra PE, et al Immune complexes containing citrullinated fibrinogen costimulate macrophages via Toll-like receptor 4 and Fcγ receptor. Arthritis Rheum 2011;63:53–62. 10.1002/art.30081 20954191 PMC3015008

[86] Asquith DL, Ballantine LE, Nijjar JS, et al The liver X receptor pathway is highly upregulated in rheumatoid arthritis synovial macrophages and potentiates TLR-driven cytokine release. Ann Rheum Dis 2013;72:2024–31. 10.1136/annrheumdis-2012-202872 23434566

[87] McInnes IB, Leung BP, Sturrock RD, et al Interleukin-15 mediates T cell-dependent regulation of tumor necrosis factor-alpha production in rheumatoid arthritis. Nat Med 1997;3:189–95. 10.1038/nm0297-189 9018238

[88] McInnes IB, Schett G Cytokines in the pathogenesis of rheumatoid arthritis. Nat Rev Immunol 2007;7:429–42. 10.1038/nri2094 17525752

[89] James EA, Rieck M, Pieper J, et al Citrulline-specific Th1 cells are increased in rheumatoid arthritis and their frequency is influenced by disease duration and therapy. Arthritis Rheumatol 2014;66:1712–22. 10.1002/art.38637 24665079 PMC4248674

[90] Ng CT, Biniecka M, Kennedy A, et al Synovial tissue hypoxia and inflammation in vivo. Ann Rheum Dis 2010;69:1389–95. 10.1136/ard.2009.119776 20439288 PMC2946116

[91] Murata K, Fang C, Terao C, et al Hypoxia-sensitive COMMD1 integrates signaling and cellular metabolism in human macrophages and suppresses osteoclastogenesis. Immunity 2017;47:66–79. 10.1016/j.immuni.2017.06.018 28723554 PMC5568808

[92] Huynh L, Kusnadi A, Park SH, et al Opposing regulation of the late phase TNF response by mTORC1-IL-10 signaling and hypoxia in human macrophages. Sci Rep 2016;6:31959 10.1038/srep31959 27558590 PMC4997257

[93] Tannahill GM, Curtis AM, Adamik J, et al Succinate is an inflammatory signal that induces IL-1β through HIF-1α. Nature 2013;496:238–42. 10.1038/nature11986 23535595 PMC4031686

[94] Miller AM, Gilchrist DS, Nijjar J, et al MiR-155 has a protective role in the development of non-alcoholic hepatosteatosis in mice. PLoS One 2013;8:e72324 10.1371/journal.pone.0072324 23991091 PMC3749101

